# Prospective cohort study on mesh shrinkage measured with MRI after laparoscopic ventral hernia repair with an intraperitoneal iron oxide-loaded PVDF mesh

**DOI:** 10.1007/s00464-017-5987-x

**Published:** 2017-12-21

**Authors:** Filip Muysoms, Roel Beckers, Iris Kyle-Leinhase

**Affiliations:** 1 Department of Surgery, Maria Middelares Hospital, Buitenring Sint-Denijs 30, 9800 Ghent, Belgium; 2 Department of Radiology, Maria Middelares, Ghent, Belgium

**Keywords:** Ventral hernia, Laparoscopy, Intraperitoneal mesh, Shrinkage, Magnetic resonance imaging, PVDF

## Abstract

**Background:**

Current data on shrinkage of intraperitoneal meshes come mainly from animal studies. High-quality human data in prospective studies are scarce.

**Methods:**

We used the ability to visualize intraperitoneal PVDF meshes enhanced with iron particles (DynaMesh IPOM visible) with magnetic resonance imaging (MRI) to determine the amount of shrinkage between 1 and 13 months postoperatively. All measurements of the width, length, and surface area of the mesh were performed with a standardized methodology independently by four radiologists blinded for the timing of the MRI.

**Results:**

Of the 15 patients undergoing laparoscopic ventral hernia repair, 13 patients received an MRI both at 1 and at 13 months. Evaluation of inter-rater reliability between the radiologists showed intra-class correlations of 0.95 (95% CI 0.92–0.98) for the width, 0.96 (95% CI 0.93–0.98) for the length, and 0.99 (90% CI 0.99–1.00) for the surface area of the mesh. The change between measurement at implantation and 1-month MRI was − 0.7 cm (*P* = 0.023; − 3.6%) for the width and − 1.9 cm (*P* = 0.001; − 7.2%) for the length. The change between 1 and 13 months was − 0.06 cm (*P* = 0.74; shrinkage = 0.3%) for the width, − 0.12 cm (*P* = 0.56; shrinkage = 0.5%) for the length, and − 4.0 cm^2^ (*P* = 0.20; shrinkage = 1.0%) for the surface area of the mesh.

**Conclusion:**

There is excellent inter-rater reliability between radiologists when measuring width, length, and surface area of visible intraperitoneal PVDF mesh with MRI. There is no significant shrinkage between 1 and 13 months of intraperitoneal PVDF mesh after laparoscopic ventral hernia repair.

## Background and rationale

It is believed that meshes used for intraperitoneal laparoscopic ventral hernia repair shrink to a variable extent after implantation [1]. It is therefore considered important to have adequate overlap of the mesh beyond the hernia defect [2]. Data on mesh shrinkage are mainly deduced from animal studies. Frequently, small animals like rats or rabbits have been used and the size of the implanted mesh has been small [3–6]. It is legitimate to ask if and how these results should be interpreted to estimate the shrinkage of mesh after clinical use in humans. Usage of a porcine animal model is limited by the rapid growth of these animals, making it difficult to perform studies with follow-up of longer than a few months [7]. At the University of Aarhus in Denmark, Paul Wara and Hans Friis-Andersen have developed an animal model of laparoscopic ventral hernia repair in sheep, which allows implantation of larger mesh sizes and have follow-up for 12–18 months [8, 9]. This model was first used to compare coated polyester mesh (Parietex™ Composite, Covidien) with composite PVDF-polypropylene mesh (DynaMesh®-IPOM, FEG Textiltechnik) and identified a shrinkage of 41 and 20%, respectively [8]. In a more recent study, several anchoring devices for mesh fixation were compared and it was concluded that the amount of shrinkage depended not only on the mesh properties, but also on the anchoring device used [9].

Limited human data on shrinkage of intraperitoneal meshes are available and most have a high risk of bias due to the retrospective nature. Three retrospective studies evaluated the shrinkage, defined as the decrease in transverse diameter of intraperitoneal ePTFE mesh (DualMesh™, WL Gore & Ass), which can be visualized on CT scan and found it to be 7.5, 6.7, and 10.6%, respectively [10–12].

Beldi et al., as part of a randomized clinical trial comparing laparoscopic ventral hernia repair using a coated polypropylene mesh (Parietene™ Composite, Covidien) either with or without the use of transfacial sutures, measured the mesh surface area with titanium clips applied to the margin of the mesh and visualized with postoperative abdominal X-ray [13]. Significantly higher shrinkage of the transverse diameter was documented (3.1 vs. 0.1%; *P* = 0.018) if no transabdominal sutures were used.

Incorporating iron oxide particles in meshes allows their precise visualization on postoperative magnetic resonance imaging (MRI) examination, thereby allowing in vivo measurement of mesh dimensions at different time points during follow-up [14, 15]. A study in ten patients using MRI to visualize intraperitoneal DynaMesh IPOM at 1 day postoperative and at 3 months showed a decrease in measured mesh surface area compared to the calculated mesh surface area at implantation of 15.6 and 19.1%, respectively [16].

### Objectives

The objective is to evaluate in a clinical setting the shrinkage, defined as the decrease of mesh surface area, between 1 and 13 months after implantation of intraperitoneal iron oxide-impregnated PVDF mesh measured with MRI. Moreover, we want to evaluate the inter-rater reliability among radiologists with regard to measurement with MRI.

## Methods

### Study design

The study is a prospective single-center observational cohort study. The study report was written in accordance with the STROBE statement (Strengthening the Reporting of Observational Studies in Epidemiology) [17].

### Setting

The study was performed at Maria Middelares Hospital in Ghent, Belgium. All operations were performed by a single surgeon experienced in laparoscopic ventral hernia repair (FM). All postoperative MRIs were coordinated by one radiologist dedicated to abdominal wall imaging (RB). The study was approved by the ethical committee at Maria Middelares Hospital Ghent with the trial number PB/nm/2013.031. Patients were invited to a combined clinical assessment and MRI imaging as an outpatient at 1 and 13 months postoperatively. The study protocol was registered at ClinicalTrials.gov (NCT02177214) before the start of the study on June 26, 2014 with the acronym IMAP study.

### Participants

#### Inclusion criteria

Individuals who were scheduled for a laparoscopic repair of a midline ventral hernia (European Hernia Society classification, M2–M3–M4 hernias only) [18], agreed to take part in the study, and signed the informed consent comprised the study sample.

#### Exclusion criteria

The following constituted our exclusion criteria: patients below 18 years, lateral hernias (L1–L4), subxiphoid hernias (M1), suprapubic hernias (M5), emergency surgery, clean-contaminated or contaminated procedures, ASA score > 4, pregnancy, life expectancy below 2 years, refusal to sign informed consent, contra-indications for MRI (implanted electrical devices, not MRI-compatible heart valves, large tattoos, large metal implants in the region of interest), and patients with claustrophobia.

### Follow-up

Patients were invited to an outpatient clinical follow-up at 1 and 13 months by the surgeon (FM) and were questioned for abdominal complaints and all adverse events since the last contact. Patients were clinically evaluated in supine and upright position, both at rest and with Valsalva maneuver. They were also invited to complete the Quality of Life questionnaire of the European Registry of Abdominal Wall Hernias (EuraHS QoL score) [19]. An MRI scan was scheduled immediately after the clinical exam.

#### Surgical technique

Patients were operated under general anesthesia with the previously described laparoscopic technique [20]. All patients were treated with an intraperitoneal PVDF meshes enhanced with iron particles (DynaMesh® IPOM visible, FEG Textiltechnik, Aachen, Germany) and fixation was performed with the double crown technique, using absorbable tacks (Securestrap®, Ethicon, Johnson & Johnson, Somerville, NJ, USA). No transabdominal fixation sutures were used.

DynaMesh® IPOM is a composite mesh specifically designed for intraperitoneal hernia repair. It is composed of 88% polyvinylidene fluoride (PVDF) monofilament on the visceral side and 12% polypropylene monofilament at the parietal side. The parietal side promotes ingrowth of the mesh to the abdominal wall and the PVDF layer on the visceral side forms a barrier to the intestines. The mesh pore size is in 80% > 1.0 mm, with an effective porosity of 41%. According to the classification by Amid et al. Dynamesh IPOM classifies as a macroporous mesh [21] and according to the classification of Klinge et al. as a large pore mesh [22].

### Imaging technique

The examination was performed with the patient in prone and feet first position using the table-integrated posterior coil of a 1.5 T magnetic resonance full digital scanner (Philips Ingenia CX). The study protocol takes about 10–12 min and consists of coronal and axial T2, sagittal in-phase FFE, coronal T1 TFE, and coronal 3D T1 non-fatsat. All sequences were performed in free breathing mode increasing patients comfort during the investigation. All images were sent to our institutional PACS system (AGFA Impax 6.5.2.657, AFGA Healthcare NV, Mortsel Belgium).

### Variables

All patient data, operative data, and postoperative data were entered in the prospective EuraHS online database [19]. Meshes were measured before implantation in their widest (horizontal) and longest (vertical) dimension. The “negative” mesh remnants remaining after cutting the mesh to the required size were kept on file for comparison to the MRI measurement if warranted.

### Data measurement

**Fig. 1:**
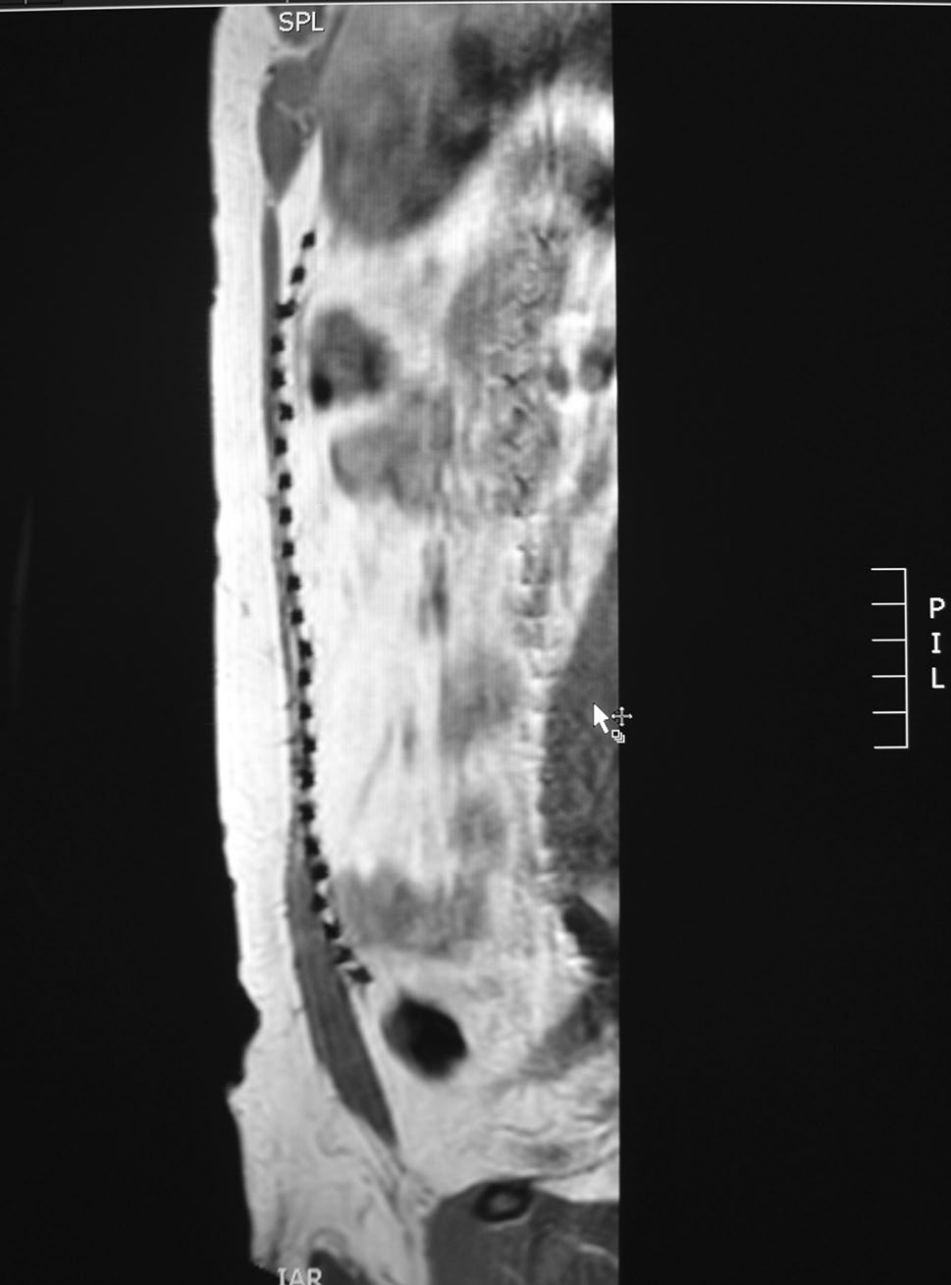
Native sagittal IF FFE (5 mm slice thickness, TE/TR 4.6/329 ms, FA 80°) MRI images after laparoscopic intraperitoneal ventral hernia repair with an iron oxide-loaded PVDF mesh

The position of the mesh was evaluated on axial, coronal, and sagittal images. Complications such as seroma, hematoma, or recurrence were excluded using the T2 and T1 images. Morphology and position of adjacent small bowel loops were evaluated to exclude adhesions of small bowel dilatation (Fig. 1). Measurements were performed on sagittal IF FFE series. All patients received sagittal IF FFE on their first and second scan. A 3D T1 low flip angle sequence was included in their second investigation. For blinding, each independent radiologist received a folder in PACS containing the anonymous and randomly ordered scan series.

### Quantitative variables

**Fig. 2:**
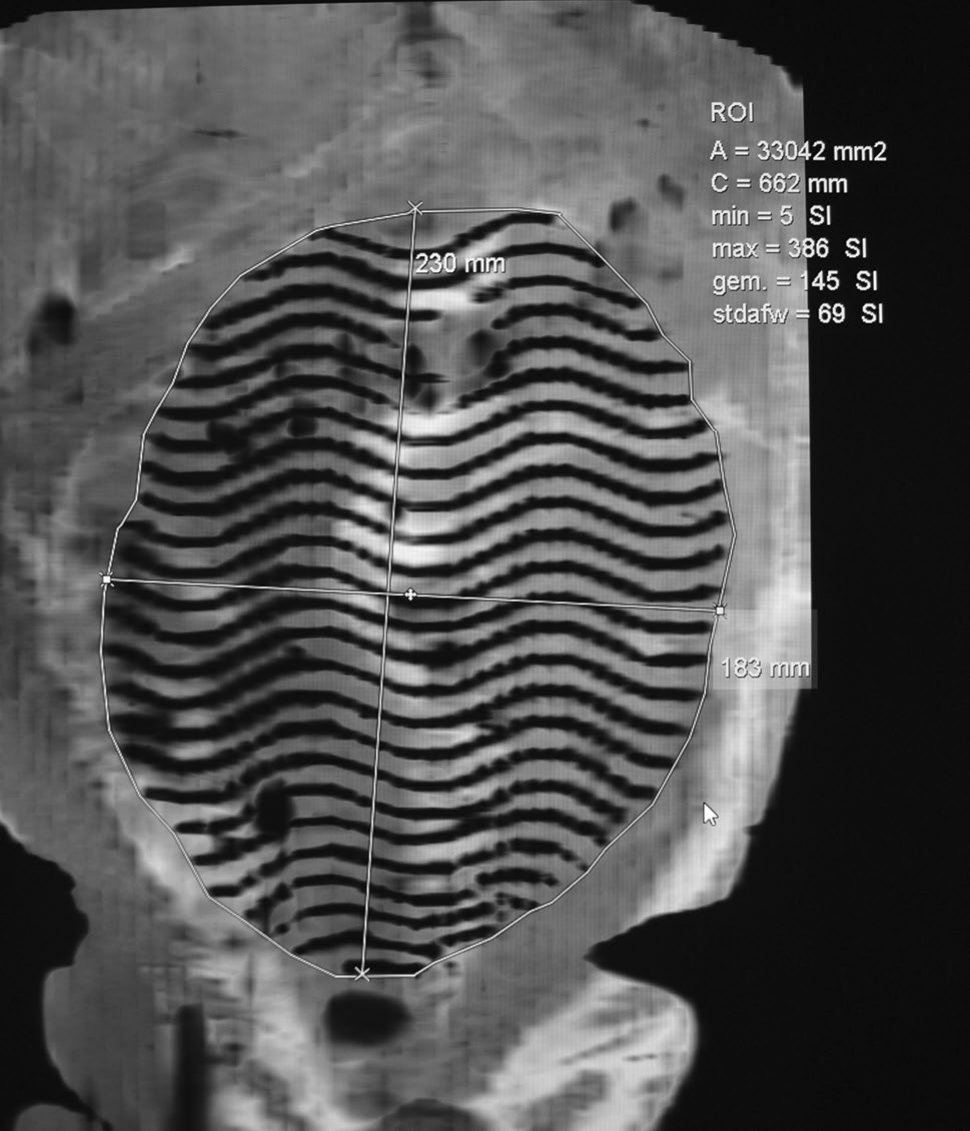
Mesh surface area was measured on a reconstructed coronal 30-mm-thick MINIP (minimal intensity projection) after loading the sagittal FFE into an MPR viewer (IMPAX Volume View) followed by drawing the mesh contour along the MR visible wires. Drawing this contour resulted in projected surface area (mm^2^) and projected circumference (mm). Based on the drawn contour, maximal orthogonal diameters (mm) were measured

A 30-mm-thick MINIP (minimal intensity projection) was created in the coronal plane after loading the sagittal IF FFE sequence in an MPR viewer (IMPAX Volume View) followed by drawing the mesh contour along the MR visible wires (Fig. 2). Drawing this contour resulted in projected surface area (mm^2^) and projected circumference (mm). Finally, based on the drawn contour maximal orthogonal diameters (mm) were measured. From the four independent measurements of each scan, the mean value of each variable was calculated as the final outcome.

Quality of Life assessment with the EuraHS QoL instrument was quantified as previously described and presented as median values with interquartile range [23].

### Bias

To minimize the risk of bias of the measurements taken of the MRI scans, the radiologists were blinded to the patients’ identity, the initial measurements of the implanted mesh, and wether the scan was after 1 or 13 months. Moreover, the scans were reviewed in a random sequence avoiding sequential measurements of scans from the same patient.

### Study size

Because no data on mesh shrinkage of implanted intraperitoneal PVDF meshes in humans were available at the start of the study, a sample size of 15 patients was empirically chosen as being large enough to evaluate the mesh shrinkage adequately and small enough to be performed within a reasonable timeframe and within the available budget.

### Statistical methods

The statistical methodology was chosen and performed by an independent statistician. The inter-rater reliability between the four radiologist was estimated by intra-class correlations [ICC (2,4); 95% CI] for the width (cm), the length (cm), and the surface area (cm^2^) of the mesh using all 27 available MRI investigations. Inter-rater reliability is considered *poor* (ICC < 0.40), *fair* (ICC 0.40–0.59), *good* (ICC 0.60–0.74), or *excellent* (ICC 0.75–1.00). Additionally, the ICC was determined separately for the 14 MRI investigations at 1 month and the 13 MRI investigations at 13 months.

The change in the width, length, and surface area of the mesh between 1 and 13 months was estimated as the mean value and standard deviation (SD). A paired *T* test was used with significance being demonstrated by a *P* value < 0.05. Additionally, marginal means and standard error (SE) were estimated using a repeated measures model. Results were reported with a graph using surface area at 1 month (cm) versus change in surface area between 1 and 13 months (cm).

The change in the width and length of the mesh between the measurements at implantation and the MRI measurements at 1 month was estimated as the mean value and SD. A paired *T* test was again used for analysis with significance being demonstrated by a *P* value < 0.05. All statistical analyses were undertaken using SAS statistical software (release 9.4).

## Results

### Participants

**Fig. 3:**
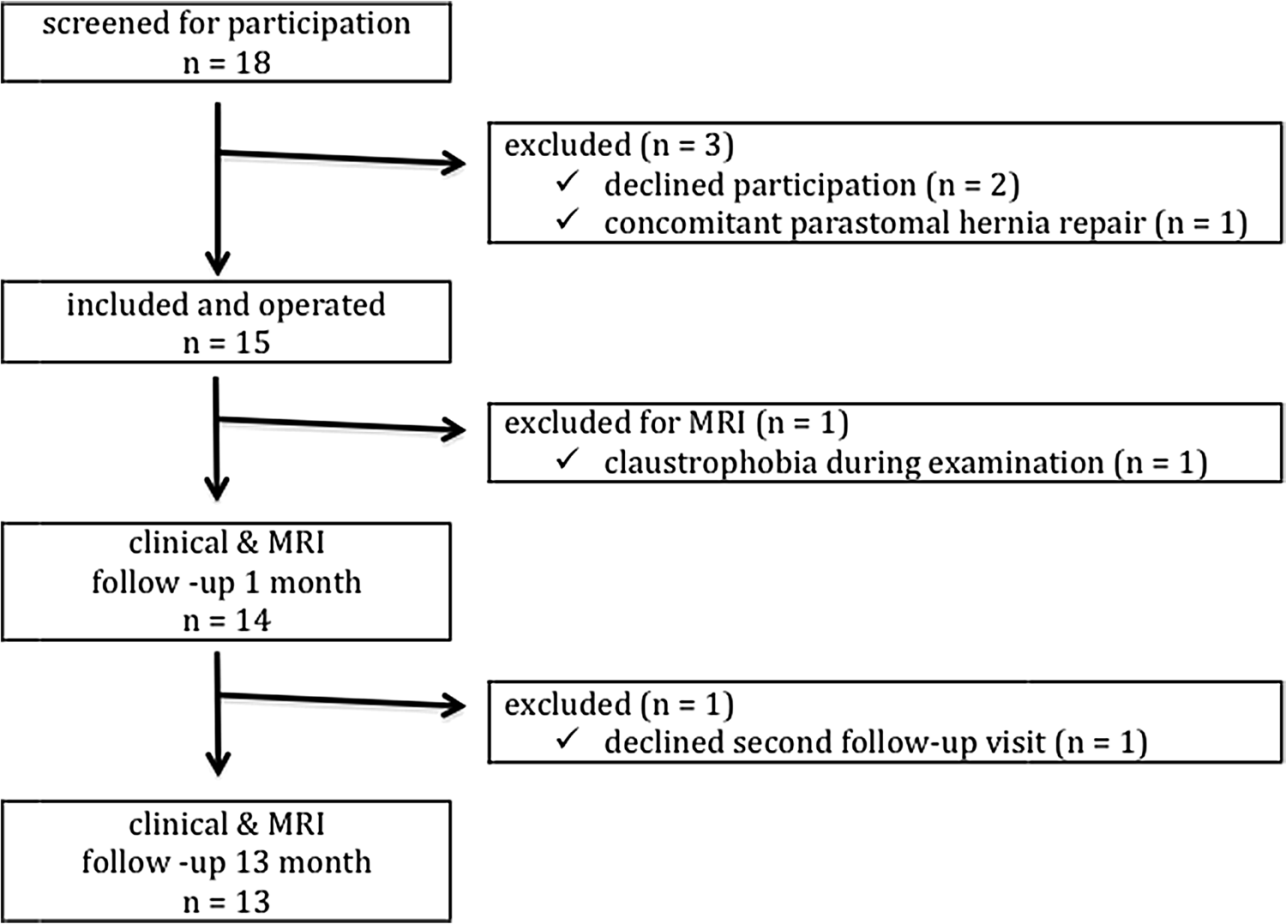
Study flow diagram of a prospective study on laparoscopic ventral hernia repair with an iron oxide-loaded PVDF mesh to evaluate the mesh shrinkage between 1 and 13 months after implantation

A study flow diagram is shown in Fig. 3. During the screening period from June 2014 to August 2015, 18 eligible patients were invited to participate in the study. Two patients declined participation and one patient was excluded intra-operatively as a concomitant parastomal hernia was identified and repaired with a modified Sugarbaker technique. Of 15 patients entered in the study, 13 were evaluated according to protocol with two postoperative MRI scans. One patient became anxious during the procedure and refused further MRI evaluation. One patient declined participation for the second clinical examination and MRI at 13 months. He reported no problems during the telephone contact to plan the follow-up visit.

### Descriptive data

Patient characteristics, intra-operative data, and postoperative clinical outcome are shown in Table 1. All hernias were midline, either primary (*n* = 8) or incisional (*n* = 7), with a mean width of 3.6 cm and a mean length of 5.1 cm. The hernia defect was closed in seven patients with a barbed suture, while in smaller hernia defects were not closed. During follow-up, two patients developed an episode of small bowel obstruction needing hospitalization. In one patient, this resolved spontaneously and in one patient a laparoscopic release of adhesions to the mesh was needed to resolve the obstruction.

**Table 1:** Patient characteristics, intra-operative data, and postoperative clinical outcome of a prospective study on laparoscopic ventral hernia repair with an iron oxide-loaded PVDF mesh to evaluate the mesh shrinkage between 1 and 13 months after implantation

	*n*/*N* or mean	Range or %
Patient demographics		
Male gender (*n*/*N*)	13/15	
Age (mean/years)	53	31–72
Patient variables		
Mean BMI (kg/m^2^)	29	22–37
Smoker	5/15	33%
Diabetes	1/15	7%
Previous other hernia repair	3/15	20%
Hernia variables		
Primary epigastric	6/15	40%
Primary umbilical	2/15	13%
Incisional hernia	7/15	47%
Width of the hernia (cm)	3.6	1.5–7.2
Length of the hernia (cm)	5.1	0.7–18.3
Operative variables		
Duration of surgery (min)	68	55–80
Defect closure	7/15	47%
Width of the mesh (cm)	20	17–24
Length of the mesh (cm)	27	21–30
Operative complications	3/15	20%
Abdominal wall bleeding	1	
Prolonged ileus	1	
Constipation	1	
Hospital stay (days)	2.5	1–7
Follow-up at 1 month		
Abdominal wall pain needing medication	2/15	
Small bowel obstruction with readmission	1/15	
Seroma	0/15	
Surgical site infections	0/15	
Recurrence	0/15	
Reoperation	0/15	
Follow-up at 13 month		
Small bowel obstruction needing reoperation	1/15	
Seroma	0/14	
Surgical site infections	0/14	
Recurrence	0/14	

### Outcome data

The outcome data of our study are shown in Table 2. The inter-rater reliability reported as the intra-class correlation was excellent (all mean ICC ≥ 0.94) for the three measurements: width, length, and surface area of the mesh. The changes between measurements at implantation, at 1, and at 13 months are shown. There is a significant decrease in width (− 0.7 cm; *P* = 0.023) and the length (− 1.9 cm; *P* = 0.001) at the 1-month measurement compared to the measurement at implantation. Between 1 and 13 months, no significant change in the width (− 0.06 cm; *P* = 0.740), the length (− 0.12 cm; *P* = 0.565), or the surface area (− 4.0 cm^2^; *P* = 0.200) was noted.

**Table 2:** Outcome data of a prospective study on laparoscopic ventral hernia repair with an iron oxide-loaded PVDF mesh to evaluate the mesh shrinkage between 1 and 13 months after implantation with MRI measurement

	Intra-class correlation^a^			
	*N*	Width	Length	Surface area
All MRI scans	27	0.95 (0.92–0.98)	0.96 (0.94–0.98)	0.99 (0.99–1.00)
MRI at 1 month	14	0.95 (0.89–0.98)	0.99 (0.97–1.00)	0.99 (0.99–1.00)
MRI at 13 months	13	0.96 (0.91–0.99)	0.94 (0.86–0.98)	0.99 (0.99–1.00)

	Change between measurements at implantation and MRI at 1 month (*N* = 14 patients)				
	Implantation	MRI at 1 month	Difference	Significance	Change (%)
	Mean (SD)	Mean (SD)	Mean (SD)	*P* value	
Width (cm)	19.9 (1.9)	19.1 (1.3)	− 0.7 (0.3)	0.023	− 3.6
Length (cm)	26.2 (2.6)	24.3 (2.1)	− 1.9 (4.5)	0.001	− 7.2

	Change between MRI at 1 month and MRI at 13 months (*N* = 13 patients)				
	MRI at 1 month	MRI at 13 months	Difference	Significance	Change (%)
	Mean (SD)	Mean (SD)	Mean (SD)	*P* value	
Width (cm)	19.1 (1.4)	19.0 (1.3)	− 0.06 (0.59)	0.740	− 0.3
Length (cm)	24.4 (2.2)	24.2 (2.0)	− 0.12 (0.74)	0.565	− 0.5
Surface area (cm^2^)	380 (44)	376 (43)	− 4.0 (10.5)	0.200	− 1.0

*N* number of MRI scans available, *MRI* magnetic resonance imaging, *Mean (SD)* mean (standard deviation)

^a^Intra-class correlation = ICC (2,4) (95% CI)

### Main results

**Fig. 4:**
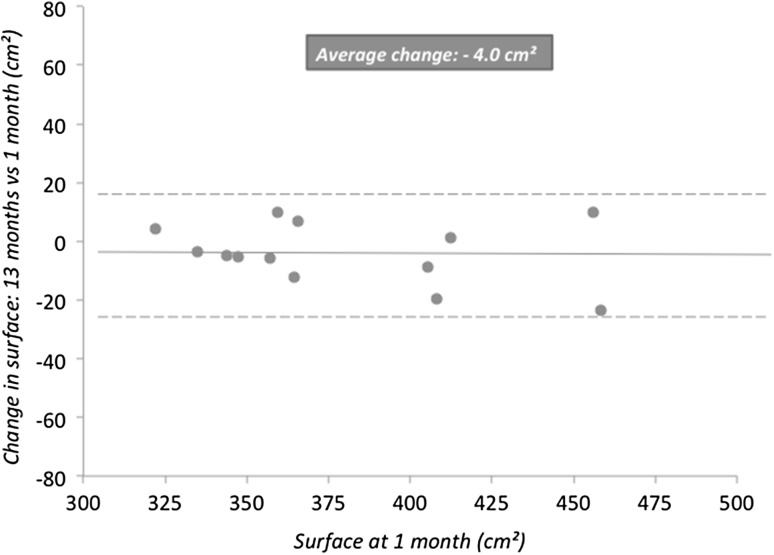
Graph for the decrease in mesh surface area (shrinkage) as determined with MRI of patients after laparoscopic ventral hernia repair with an iron oxide-loaded PVDF mesh. The change in surface area between 1 and 13 month postoperatively is plotted against the mesh surface area measured at 1 month. The full line indicates the mean decrease in mesh surface area (− 0.4 cm^2^) and the interrupted lines above and below indicate 2 × SD (standard deviation) from the mean (2 × 10.5 cm^2^)

No significant decrease in mesh surface area (shrinkage) was demonstrated between 1 and 13 months with a mean change in surface area of − 4.0 cm^2^ (− 1%). As illustrated in Fig. 4, plotting the change in mesh surface area versus the surface area at 1 month, none of the patients had a shrinkage outside of the boundaries of two SD from the mean.

### Other analyses

**Fig. 5:**
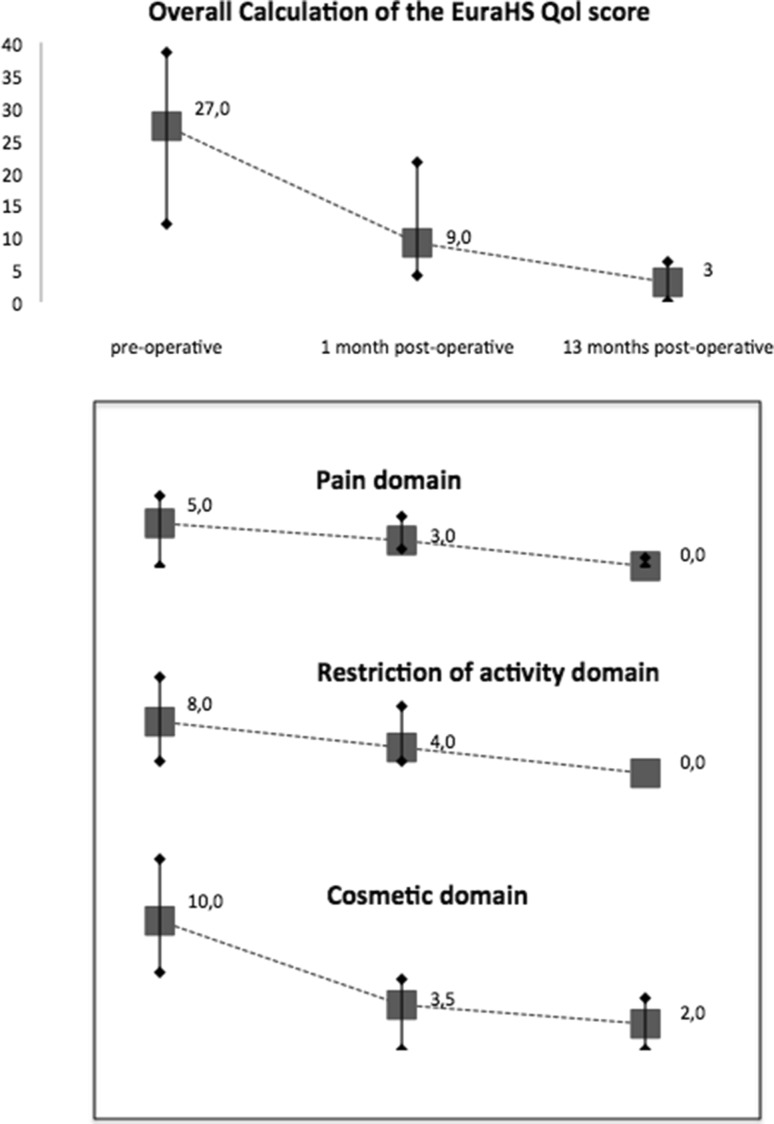
Graphic report of the Quality of Live assessment with the EuraHS QoL instrument in a prospective study on laparoscopic ventral hernia repair with an iron oxide-loaded PVDF mesh. Results are presented as median values with interquartile ranges

The assessment of Quality of Live using the EuraHS QoL instrument showed significant improvement after 1 month compared to the preoperative assessment and a further improvement at 13 months. The results are shown in Fig. 5.

## Discussion

### Key result

There was no significant shrinkage of intraperitoneal PVDF mesh between 1 and 13 months after laparoscopic ventral hernia repair.

### Limitations

The sample size of our study is not very large, but in line with many of the animal studies on mesh shrinkage of intraperitoneal meshes. Nevertheless, since the mean changes in mesh width, length, and surface area were negative, it is likely that a much larger sample size might demonstrate shrinkage to a statistically significant level even with small absolute degrees of shrinkage. On the other hand, the clinical relevance of such a small absolute decrease of mesh surface area is probably low.

We have chosen patients with ventral midline hernias only as this seemed the best population to obtain reproducible and reliable measurements. It is possible that in lateral, subxiphoid, or suprapubic hernias a higher degree of mesh shrinkage would be observed because of the less constant flat positioning of the mesh.

### Interpretation

The degree of shrinkage of PVDF intraperitoneal mesh in our study is much lower than the 20% shrinkage found in a study on sheep by Zinther et al. [8]. They calculated the shrinkage as the decrease in mesh size between the implanted mesh and the mesh measured up to 18 months postoperatively. Our study notes that there seems to be a discrepancy in mesh size from implantation to 1 month postoperatively. We think this can be attributed to the incomplete flat alignment of the mesh against the abdominal wall during surgery, more than to actual shrinkage after implantation. Moreover, although sheep have a lower growth rate than pigs, the growth was still significant during the study period (25% of weight increase during 12 months), which is not the case for adult humans. They also reported that after the initial shrinkage during the first 3 months no further significant shrinkage was noted.

Comparing our results to the other human study using visible intraperitoneal PVDF mesh, we also found a lower shrinkage rate [16]. Köhler et al. found at 3 months a shrinkage of the mesh surface area of 19% compared to the implantation size of the mesh. But their baseline MRI on the first postoperative day already demonstrated shrinkage of 15.6%, and thus the majority of the mesh size discrepancy at 3 months should be attributable to the immediate decrease after implantation, probably due to wrinkling of the mesh. The decrease in mesh size in their study between the MRI on postoperative day 1 and at 3 months was only 4.2%.

We think our study shows that after implantation and a period of ingrowth of the mesh during the first month, no additional significant decrease in mesh size is present for intraperitoneal PVDF mesh fixed with a double crown of absorbable tacks. This finding was consistently seen in all the patients, with no outliers demonstrating significant shrinkage. Our study does not support the concept that an individual biological reaction of the patient to the implanted material might cause a highly variable amount of shrinkage between patients for this type of mesh in combination with this type of fixation.

Our findings should not be compared with human studies using intraperitoneal ePTFE mesh due to differences in mesh material, different methodology in mesh size measurement, different definitions for mesh shrinkage, and the high level of bias in most of these studies due to their retrospective nature [10–13].

In our opinion, mesh shrinkage should be determined on measurements of mesh surface area rather than only on the width or the length of the mesh. Mesh shrinkage between two times points (*T*_1_ and *T*_2_) should be defined as “The percentage of decrease in mesh surface area between two points in time after implantation of the mesh.” The method of measurement or calculation of the mesh surface area should be similar for both time points. Comparing a measured mesh surface area after implantation with the calculated mesh surface area at implantation is not considered adequate to detect shrinkage, but it is rather the result of the three-dimensional wrinkling and folding of the mesh during implantation and during release of the pneumoperitoneum.

We propose the following formula for calculating mesh shrinkage, where *T*_1_ and *T*_2_ are postoperative selected time points.

$${\text{Mesh shrinkage between }}{{{T}}_1}{\text{ and }}{{{T}}_2}\left( \% \right)=100 - \frac{{{\text{surface area at }}{{{T}}_2}\left( {{\text{c}}{{\text{m}}^2}} \right)~ \times 100}}{{{\text{surface area at }}{{{T}}_1}\left( {{\text{c}}{{\text{m}}^2}} \right)}}.$$

Comparing the mesh surface area measured postoperatively to the mesh surface area calculated at implantation and estimating the decrease in effective surface area of the mesh after implantation is interesting to determine the amount of overlap that can be accomplished. But we do not think that this should be labeled as mesh shrinkage.

### Generalizability

Our findings are only applicable to intraperitoneal PVDF mesh fixed with Securestrap absorbable tacks. Other studies have shown that the amount of shrinkage is dependent on the mesh type [8]. Meshes have a large number of variables including the polymer, pore size, weave or knitting details, elasticity, and anti-adhesive coating. These factors may have a significant influence on the rate of shrinkage. Other studies have shown that the type of fixation also has an important impact on the shrinkage [9, 13]. Beldi et al. found a significantly lower decrease in width of the mesh when transabdominal fixation sutures were added to the tacks fixation [13]. In addition, the mesh position might be important, and our results for intraperitoneal PVDF mesh cannot automatically also be used to estimate the shrinkage rate after retro-muscular mesh placement.

We had two patients with postoperative small bowel obstruction during follow-up, one of them needing a reoperation. The composite PVDF-polypropylene mesh (DynaMesh®-IPOM, FEG Textiltechnik) has demonstrated favorable long-term results after intraperitoneal placement in a prospective observational study by Berger et al. in 344 patients with a mean follow-up of 24 months [24]. Other authors have published some concerns with the intraperitoneal use of this mesh in a much smaller patient cohort of 29 patients [25]. A more recent prospective study by Sommer et al. demonstrated a 6% reoperation rate after a median follow-up of 36 months, and although some reoperations were because of adhesions, the majority were reoperations for a symptomatic recurrence [26]. In a retrospective study on 88 patients undergoing laparoscopic ventral hernia repair, Tandon et al. compared 62 Parietex™ Composite meshes and 26 DynaMesh® IPOM meshes [27]. With a median follow-up of 53.6 months, they found a recurrence rate of 12.9 and 3.8%, respectively, for Parietex™ Composite and DynaMesh® IPOM. DynaMesh® IPOM was associated with a significant higher incidence of intestinal obstructions to adhesions. Our study is too small to add significant knowledge about the frequency of adhesion formation and small bowel obstruction after intraperitoneal PVDF-polypropylene mesh.

## Other information

### Study registration

The study protocol was registered at ClinicalTrials.gov (NCT02177214) before the start of the study on June 26, 2014 with the acronym IMAP study.
